# C3a/C3aR Affects the Propagation of *Cryptosporidium parvum* in the Ileum Tissues of Mice by Regulating the Gut Barrier, Cell Proliferation, and CD4^+^ T Cell Main Effectors

**DOI:** 10.3390/ani13050837

**Published:** 2023-02-24

**Authors:** Xin Yang, Xuemei Wu, Shuang Huang, Qian Yao, Xi Chen, Junke Song, Yingying Fan, Guanghui Zhao

**Affiliations:** Key Laboratory of Ruminant Disease Prevention and Control (West), College of Veterinary Medicine, Northwest A&F University, Yangling 712100, China

**Keywords:** *Cryptosporidium parvum*, C3a/C3aR signaling, propagation, intestinal barrier function, cell proliferation, CD4^+^ T cell-related cytokines

## Abstract

**Simple Summary:**

The complement system plays important roles in both innate and adaptive immunity. The present study explored the function of host C3a/C3aR signaling during *Cryptosporidium parvum* infection, and found that the C3a/C3aR signaling likely affected the propagation of *C. parvum* in mouse ileum tissues by regulating the gut barrier, cell proliferation and CD4^+^ T cell main effectors.

**Abstract:**

*Cryptosporidium parvum* is an important zoonotic protozoon that threatens the health of humans and animals, but the interaction mechanisms between *C. parvum* and hosts are poorly understood. Our previous study indicated that the expression levels of C3a and C3aR were up-regulated in mice during *C. parvum* infection, but the mechanisms of C3a/C3aR signaling during *C. parvum* infection have not been elucidated. In the present study, an optimized BALB/c suckling mouse model infected with *C. parvum* was used to explore the function of C3a/C3aR signaling during *C. parvum* infection. The expression levels of C3aR in the ileum tissues of mice infected with *C. parvum* were analyzed using real-time PCR, Western blot and immunohistochemistry. The mRNA levels of the *Cryptosporidium 18S rRNA* gene, tight junction proteins (*zo-1*, *claudin 3*, and *occludin*), intestinal stem cell marker *lgr5*, cell proliferation marker *ki67*, Th1 cell-related cytokine *ifn-γ*, and Treg cell-related cytokine *tgf-β* in mouse ileum tissues were analyzed by real-time PCR. The pathological injury of ileal mucosa was examined by histopathology analysis. The mRNA expression levels of *Cryptosporidium 18S rRNA* gene were significantly up-regulated in the ileum tissues of C3aR-inhibited mice during *C. parvum* infection. Meanwhile, histopathology analysis of ileal mucosa in mice showed that inhibition of C3aR significantly aggravated the changes in villus length, villus diameter, mucosal thickness and the ratio of villus length to crypt depth during *C. parvum* infection. Further studies found inhibition of C3aR aggravated the down-regulation of *occludin* at most time points during *C. parvum* infection. The mRNA levels of *ki67* and *lgr5* in the ileum tissues of mice infected with *C. parvum* were significantly down-regulated. Inhibition of C3aR significantly down-regulated the mRNA expression levels of *lgr5* at most time points, but significantly up-regulated the mRNA expression levels of *ki67* at most time points. The mRNA expression levels of *ifn-γ* and *tgf-β* were significantly up-regulated and down-regulated in the ileum tissues of mice infected with *C. parvum*, respectively. However, inhibition of C3aR significantly increased the mRNA expression levels of *ifn-γ* and *tgf-β* in the ileum tissues of mice infected with *C. parvum*. Taken together, C3a/C3aR signaling could possibly affect the propagation of *C. parvum* in mouse ileum tissues by regulating the gut barrier, cell proliferation and CD4^+^ T cell main effectors, which would contribute to our understanding of the interaction between *Cryptosporidium* and hosts.

## 1. Introduction

*Cryptosporidium* spp. are important diarrheal pathogens threatening the health of humans and hundreds of animals [1,2]. *Cryptosporidium* spp. are primary diarrheal pathogens in dairy cattle, with over 20% of young animals infected with the pathogens [3,4]. Meanwhile, children and immunosuppressed populations are highly susceptible to *Cryptosporidium* [5]. *Cryptosporidium* spp. have been recognized as one of five major diarrheal pathogens in children under two years of age in developing countries [5], and have led to hundreds of outbreaks of diarrhea in industrialized nations [6]. Nitazoxanide is the only FDA-approved drug in the treatment of human cryptosporidiosis, but it is almost useless in children and immunosuppressed populations [7]. Therefore, hitherto, there are still no effective strategies for the treatment of cryptosporidiosis in humans and animals. Knowledge on the interaction between *Cryptosporidium* and hosts can shed the light on the discovery of novel targets for the development of vaccines and drugs, providing resources for the prevention and control of cryptosporidiosis in humans and animals.

Previous studies have found that both innate and acquired immune responses played important roles in the early defense and late elimination of *Cryptosporidium* [8]. During the early stages of acute infection, innate immune response participates in the host defense against *Cryptosporidium* invasion by monitoring immunocytes, cytokines, chemokines and complement molecules [8,9,10,11,12]. More importantly, activated innate immune response can trigger and regulate acquired immune responses [10]. Acquired immune responses are necessary to eliminate *Cryptosporidium*, especially for the cellular immune response dominated by CD4^+^ T cells [8,13,14,15]. As the main components of innate immune response, complement molecules can play roles in dissolving pathogens, regulating phagocytosis, and mediating inflammation, immune adhesion and cytotoxicity in infected areas through the classical pathway, alternative pathway and lectin pathway [16]. Moreover, activated complement components (e.g., C3d, complement receptor CD21/35 and C5aR) can be involved in the regulation of inflammation and acquired immune response dominated by CD4^+^ T cells, and linked with the innate immunity with acquired immunity [17,18,19,20].

Complement C3 hydrolyzes to form anaphylaxis toxin C3a after the activation of the complement system, and C3a can bind to its specific receptor C3aR on the surface of cells; it then functions in inflammation, intestinal injury repairment, and CD4^+^ T cell differentiation and development [21,22,23]. Our previous studies indicated the up-regulation of C3a and C3aR in mice during *C. parvum* infection, but the role of C3a/C3aR signaling during *Cryptosporidium* infection was still unknown [24]. Therefore, the present study applied an optimized BALB/c mouse model infected with *C. parvum* to explore the function of C3a/C3aR signaling during *C. parvum* infection, which would contribute to our understanding of the interaction between *C. parvum* and hosts.

## 2. Materials and Methods

### 2.1. Parasites

The *C. parvum* IIdA19G1 strain was isolated from one pre-weaned dairy calf with diarrhea in the Shaanxi province of China, and identified based on sequence analysis of the *18S rRNA* and the *gp60* gene loci [25,26]. The strain was isolated in the lab of parasitology of Northwest A&F University and passaged in pre-weaned dairy calves in specific pathogen-free conditions. *Cryptosporidium parvum* oocysts in the faeces were purified using the Sheather’s sugar flotation technique and cesium chloride density gradient centrifugation, then stored in PBS with 100 U/mL penicillin, 0.1 mg/mL streptomycin and 0.25 μg/mL amphotericin B solutions for less than 3 months, as in the previous study [27].

### 2.2. In Vivo Infection Model

An established BALB/c suckling mouse model infected with *C. parvum* in our group [27] was optimized in the present study by increasing the concentration of sodium taurocholate from 0.1% to 1%, and the excystation time from 30 min to 1 h. Briefly, each suckling mouse of the infection group and control group was orally administrated with 1 × 10^7^ oocysts and equal volume of PBS, respectively. Suckling mice in the infection group and the control group were kept in separate cages to avoid contamination. At 1 day post infection (dpi), 2 dpi, 4 dpi, 6 dpi, 8 dpi, 9 dpi, 10 dpi, 29 dpi, 30 dpi, 35 dpi and 36 dpi, three animals were randomly selected for the isolation of ileum tissues from both control and infected groups at each time point, respectively. The above-mentioned time points and the range were selected since they represented specific milestones in the infection development. The ileum tissues collected from three animals for each group at each time point were washed in PBS and stored at −80 °C for RNA and protein isolation. Meanwhile, the ileum tissues at the peak of infection (6 dpi), based on RT-qPCR of *18S rRNA* gene in mouse ileum tissues, were also stored in 4% paraformaldehyde fix solution for histopathology analysis, and the contents of large intestine at the peak of infection were kept at 4 °C for acid-fast stain analysis. All the experiments were performed thrice.

The contents of the large intestine at the peak of infection from both the infected and control groups were analyzed using acid-fast stain to identify *C. parvum* oocysts, as previously reported [28].

Histopathology analyses of mouse ileum tissues at the peak of infection were conducted as previously reported [29]. Five slides of each mouse ileum tissue sample were randomly selected for calculating villus length, villus diameter, mucosal thickness and the ratio of villus length to crypt depth. For each slide, five complete intestinal villi with the highest length were analyzed.

Furthermore, a C3aR-inhibited BALB/c suckling mouse model infected with *C. parvum* was established by intraperitoneally injecting animals with C3aR antagonist SB290157 (MedChemExpress, Princeton, NJ, USA) at a dose of 10 mg/kg before *C. parvum* infection [30]. Collection of ileum tissue, RNA isolation and cDNA synthesis, expression level analysis of *Cryptosporidium 18S rRNA* gene, and histopathology analyses were conducted to explore the effect of C3a/C3aR signaling on the propagation of *C. parvum* in the ileum of mice.

### 2.3. Reverse Transcriptase Quantitative Polymerase Chain Reaction (RT-qPCR)

Extraction of total RNA from mouse ilea and subsequent synthesis of cDNA were the same as previous study [31]. The relative expression levels of *Cryptosporidium 18S rRNA* gene, *C3aR*, *zo-1*, *claudin 3*, *occludin*, *lgr5*, *ki67*, *ifn-γ* and *tgf-β* in mouse ileum tissues were evaluated by qPCR using SYBR Green Fast RT-qPCR Mix (ABclonal, Wuhan, China) according to the manufacturer’s recommendations and with the primers listed in Table 1. The *gapdh* gene was also included for data normalization (Table 1). Three independent experiments for tissues collected from three animals were performed, with data calculated by applying the 2^−ΔΔCt^ method to assess mRNA expression.

### 2.4. Western Blot Analysis

The protein expression levels of C3aR in mouse ileum tissues at 2 dpi and 6 dpi were analyzed by Western blotting. Briefly, the mouse ileum tissues were homogenized by grinding, and treated with 1 mL RIPA (ComWin, Beijing, China) containing 1% PMSF (Beyotime, Shanghai, China) for the isolation of proteins. Proteins were separated by SDS-PAGE analysis, and transferred to nitrocellulose membranes pre-activated with methanol. Subsequently, the membranes were blocked with TBST containing 5% non-fat milk for 2 h, and incubated with anti-C3aR antibodies (Sanying, Wuhan, China) with 1:1,000 dilution at 4 °C overnight. The membranes were washed in TBST for 5 min thrice. HRP-conjugated goat-anti-rabbit antibodies (Sangon, Shanghai, China) with 1:5,000 dilution were applied as the secondary antibodies to incubate the membranes for 1 h at room temperature. Then, the membranes were washed in TBST for 5 min thrice and treated with Super ECL Plus (Applygen, Beijing, China) for photography under an automatic gel imaging analysis system (Sage, Beijing, China).

### 2.5. Immunohistochemistry Analysis

The mouse ileum tissues at the peak of infection were analyzed by immunohistochemistry using SP-0023 Histostain^TM^-Plus Kits (MBL, Beijing, China). Briefly, paraffin sections were made from the ileum tissues at the peak of infection and subsequently incubated with anti-C3aR antibodies (Sanying, Wuhan, China) with 1:200 dilution at 4 °C overnight and HRP-conjugated goat-anti-rabbit antibodies (Sangon, Shanghai, China) for 15 min at room temperature. These were then photographed under a microscope (Olympus, Hataya, Japan). Image-Pro Plus was used to assess the distribution of C3aR on the surface of mouse ileum tissues by calculating the mean optical density value. Accumulative optical density divided by the area of cell distribution equals the mean optical density value.

### 2.6. Data Analysis

Statistical difference analysis was conducted by GraphPad Prism V.8.0 (https://www.graphpad.com/scientific-software/prism/, accessed on 7 July 2022) using Student’s *t* test and ANOVA [32]. Significant difference was identified if the *p* value was less than 0.05.

## 3. Results

### 3.1. Optimization of a Mouse Model Infected with C. parvum

**Figure 1:**
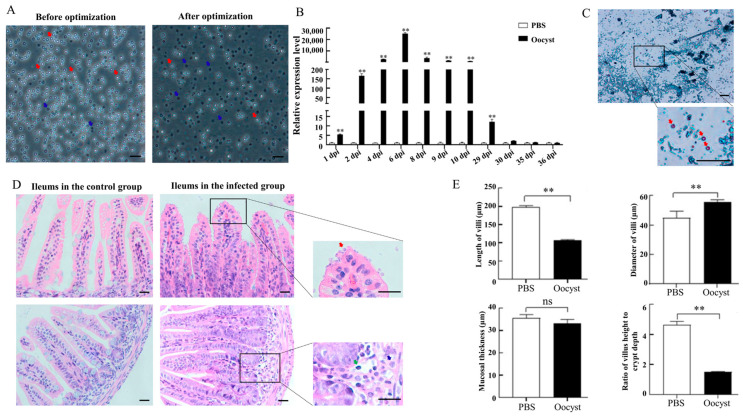
Optimization of mouse model infected with *C. parvum*. (**A**) Excystation results of *C. parvum* oocysts. Unexcysted and excysted oocysts appeared as light refractile spheres (red arrow) and dark black spheres (blue arrow), respectively. Bars = 20 μm. (**B**) qRT-PCR amplification results of *Cryptosporidium 18S rRNA* gene. ^**^ means *p* < 0.01. (**C**) Acid-fast staining results for contents in large intestines of mice infected with *C. parvum* at 6 dpi. Oocysts appeared as rosy spheres (red arrow). Bars = 20 μm. (**D**) HE staining results in ileum tissues of mice in the control and infected groups at 6 dpi. *C. parvum* in the brush border of ileum villi in infected group (red arrow) and lymphocytes (green arrow) and neutrophils (blue triangle) infiltration in the lamina propria were indicated. Bars = 20 μm. (**E**) Analysis of pathological lesions in ileum tissues of mice infected with *C. parvum* at 6 dpi. Error bars mean standard deviation with each group, ** means *p* < 0.01, and ^ns^ means *p* > 0.05.

Optimization of the excystation scheme could significantly increase the excystation rate from 40% to over 85%, as shown in Figure 1A (*p* < 0.05). Compared with the control group, the relative mRNA expression levels of *Cryptosporidium 18S rRNA* gene in the mouse ilea in *C. parvum*-infected group increased gradually and reached a peak at 6 dpi, then gradually decreased. The duration period of *C. parvum* infection in the mouse model reached 29 days (Figure 1B). Acid-fast stain analysis of the large intestine contents at 6 dpi recognized rosy oocysts in the infected group (Figure 1C). Histopathology analyses of mouse ileum tissues at 6 dpi found the existence of *C. parvum* and the infiltration of the inflammatory cells in the ileum tissues of the infected group (Figure 1D); subsequently statistical analysis indicated *C. parvum* infection significantly deceased the length of villi and the ratio of villus height to crypt depth, and significantly increased the diameter of villi (Figure 1E).

### 3.2. Expression of C3aR in Mouse Ileum Tissues during C. parvum Infection

**Figure 2:**
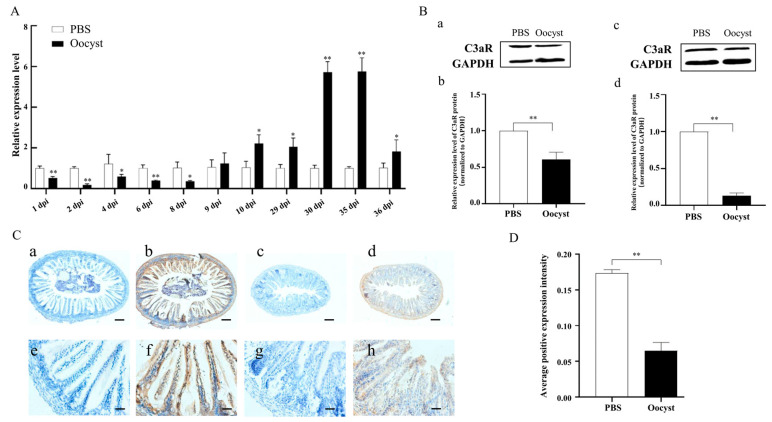
Expression of C3aR in the ilea of mice infected with *C. parvum*. (**A**) Analysis of relative expression levels of C3aR in ileum tissues of mice infected with *C. parvum*; (**B**) C3aR protein expression levels in ileum tissues of mice. a and c: Western blotting analysis at 2 and 6 dpi; b and d: statistical analysis at 2 and 6 dpi; (**C**) Immunohistochemical staining results of C3aR protein in ileum tissues (6 dpi) of mice. a and e: no primary antibody (control group); b and f: with primary antibody (control group); c and g: no primary antibody (infected group); d and h: with primary antibody (infected group); (**D**) Statistic analysis of immunohistochemical staining results of C3aR protein. * means *p* < 0.05, ** means *p* < 0.01.

Compared with control group, the mRNA expression levels of C3aR in mouse ileum tissues of the infected group were significantly down-regulated from 1 to 8 dpi, and then up-regulated until 36 dpi (Figure 2A). Western blot analysis also indicated the down-regulation of protein levels of C3aR in mouse ileum tissues of the infected group at both 2 dpi and 6 dpi (Figure 2B, Figures S1 and S2). Further immunohistochemistry analysis indicated C3aR was majorly distributed on the surface of ileal epithelial cells at 6 dpi (Figure 2C), and subsequently statistical analysis also showed significantly decreased distribution of C3aR in mouse ileum tissues of the infected group (Figure 2D).

### 3.3. Effect of C3a/C3aR Signaling on the Propagation of C. parvum in the Ilea of Mice

**Figure 3:**
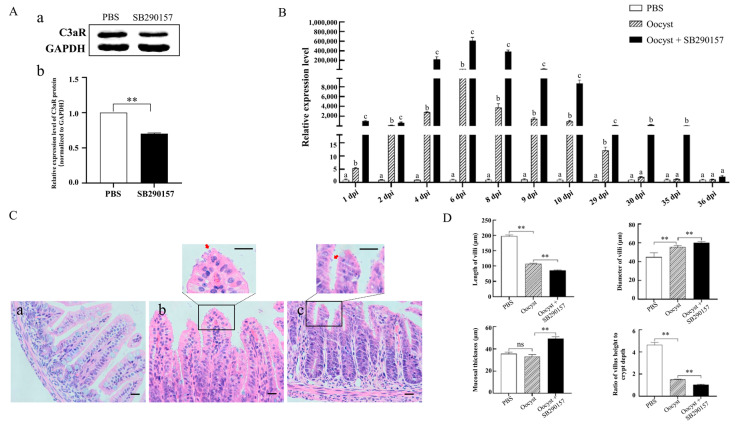
Effect of C3a/C3aR signaling on the propagation of *C. parvum* in mouse ilea. (**A**) C3aR protein expression levels in ileum tissues of mice detected using Western blotting analysis. a: Western blotting results; b: statistical analysis of Western blotting results. ** means *p* < 0.01; (**B**) Analysis of relative transcriptional expression levels of *Cryptosporidium 18S rRNA* gene in the ileum tissues of mice. The relative expression levels of *Cryptosporidium 18S rRNA* between two groups at each time point were compared, and significant differences were found between the relative expression levels of *Cryptosporidium 18S rRNA* between two groups if lowercase letters above the bars were different (*p* < 0.05); (**C**) HE staining results in ileum tissues of mice infected with *C. parvum* at 6 dpi. a/b/c indicate ilea in the control group/infected group/C3aR-inhibited group, respectively; (**D**) Analysis of pathological lesions in ileum tissues of mice infected with *C. parvum* at 6 dpi. ** means *p* < 0.01, ns means *p* > 0.05.

As shown in Figure 3A, the protein level of C3aR in mouse ileum tissues was significantly down-regulated three hours after intraperitoneal injection with SB290157 (Figure 3A and Figure S3). Further studies indicated that inhibition of mouse C3aR significantly up-regulated the relative mRNA expression levels of the *Cryptosporidium 18S rRNA* gene in mouse ileum tissues during *C. parvum* infection (*p* < 0.05). Meanwhile, the infection duration period increased from 29 days to 35 days in the C3aR-inhibited infected group (Figure 3B). Significant differences were found between the relative transcriptional expression of *Cryptosporidium 18S rRNA* among the three groups (PSB, Oocyst, and Oocyst + SB290157) at 1 dpi (F = 359.612, *p* < 0.001), 2 dpi (F = 58.443, *p* < 0.001), 4 dpi (F = 44.112, *p* < 0.001), 6 dpi (F = 215.622, *p* < 0.001), 8 dpi (F = 393.826, *p* < 0.001), 9 dpi (F = 114.706, *p* < 0.001), 10 dpi (F = 427.847, *p* < 0.001), 29 dpi (F = 41.950, *p* < 0.001), 30 dpi (F = 55.628, *p* < 0.001), 35 dpi (F = 25.905, *p* < 0.001) and 36 dpi (F = 13.106, *p* = 0.006). Histopathology analyses of mouse ileum tissues at 6 dpi found the existence of *C. parvum* in the ileum tissues in the infected group and the C3aR-inhibited group (Figure 3C). Subsequent statistical analysis indicated that *C. parvum* infection in C3aR-inhibited group further decreased the length of villi and the ratio of villus height to crypt depth, and increased the diameter of villi and mucosal thickness compared with the normal mouse in the infected group (Figure 3D), suggesting that C3aR likely alleviated the intestinal injury caused by *C. parvum*.

### 3.4. Transcriptional Expression Level Analysis of Tight Junction Proteins in the Ileum Tissues of Mice during C. parvum Infection

**Figure 4:**
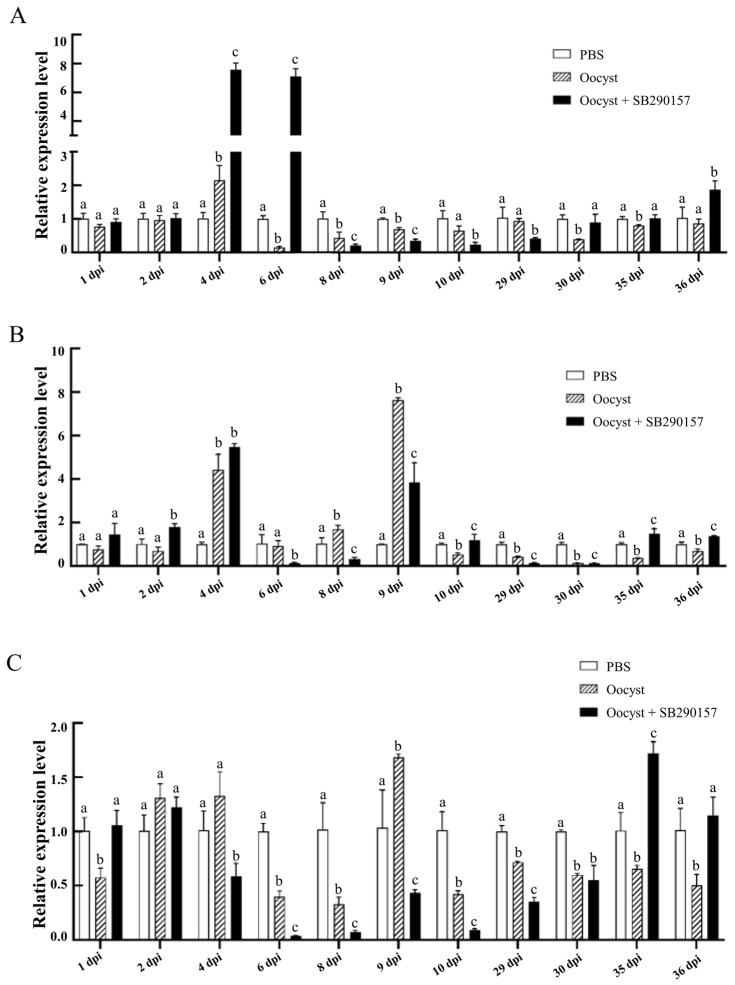
Effect of C3aR inhibition on the relative transcriptional expression of intestinal barrier-related genes in the ilea of mice infected with *C. parvum*. Analysis of the relative expression levels of the *zo-1* gene (**A**), *claudin 3* gene (**B**) and *occludin* gene (**C**) in the ileum tissues of mice. Different lowercase letters above the bars indicated significant differences (*p* < 0.05).

Significant differences were found between the relative transcriptional expression levels of the *zo-1* gene among three groups (PSB, Oocyst, and Oocyst + SB290157) at 4 dpi (F = 253.975, *p* < 0.001), 6 dpi (F = 434.805, *p* < 0.001), 8 dpi (F = 21.897, *p* = 0.002), 9 dpi (F = 164.639, *p* < 0.001), 10 dpi (F = 18.990, *p* = 0.003), 29 dpi (F = 9.539, *p* = 0.014), 30 dpi (F = 14.252, *p* = 0.005), 35 dpi (F = 7.212, *p* = 0.025) and 36 dpi (F = 14.015, *p* = 0.005), while differences at 1 dpi (F = 3.316, *p* = 0.107) and 2 dpi (F = 0.146, *p* = 0.867) were not significant. *Cryptosporidium parvum* infection significantly up-regulated the mRNA expression levels of the *zo-1* at 4 dpi and down-regulated at 6 dpi, 8 dpi, 9 dpi, 30 dpi and 35 dpi. Inhibition of C3aR led to dynamic changes in the *zo-1* gene during *C. parvum* infection, reflected by the down-regulation at 8 dpi, 9 dpi, 10 dpi and 29 dpi, and up-regulation at 4 dpi, 6 dpi, 30 dpi, 35 dpi and 36 dpi (Figure 4A).

Significant differences were found between the relative transcriptional expression of the *claudin 3* gene among three groups (PSB, Oocyst, and Oocyst + SB290157) at 2 dpi (F = 29.079, *p* = 0.001), 4 dpi (F = 89.677, *p* < 0.001), 6 dpi (F = 10.677, *p* = 0.011), 8 dpi (F = 37.588, *p* < 0.001), 9 dpi (F = 119.767, *p* < 0.001), 10 dpi (F = 13.474, *p* = 0.006), 29 dpi (F = 210.694, *p* < 0.001), 30 dpi (F = 380.076, *p* < 0.001), 35 dpi (F = 52.630, *p* < 0.001) and 36 dpi (F = 60.535, *p* < 0.001), while the difference at 1 dpi (F = 3.593, *p* = 0.094) was not significant. Compared with the control group, the mRNA expression levels of the *claudin 3* gene of mouse ileum tissues in *C. parvum*-infected group were significantly down-regulated at 10 dpi, 29 dpi, 30 dpi, 35 dpi and 36 dpi, and up-regulated at 4 dpi, 8 dpi and 9 dpi. Inhibition of C3aR led to dynamic changes in the *claudin 3* gene during *C. parvum* infection, reflected by the down-regulation at 6 dpi, 8 dpi, 9 dpi, 29 dpi and 30 dpi, and up-regulation at 2 dpi, 10 dpi, 35 dpi and 36 dpi (Figure 4B).

Significant differences were found between the relative transcriptional expression of the *occludin* gene among three groups (PSB, Oocyst, and Oocyst + SB290157) at 1 dpi (F = 15.306, *p* = 0.004), 4 dpi (F = 13.151, *p* = 0.006), 6 dpi (F = 257.643, *p* < 0.001), 8 dpi (F = 33.276, *p* = 0.001), 9 dpi (F = 29.416, *p* = 0.001), 10 dpi (F = 62.741, *p* < 0.001), 29 dpi (F = 219.563, *p* < 0.001), 30 dpi (F = 28.790, *p* = 0.001), 35 dpi (F = 67.671, *p* < 0.001) and 36 dpi (F = 13.095, *p* = 0.006), while the difference at 2 dpi (F = 4.718, *p* = 0.059) was not significant. *Cryptosporidium parvum* infection significantly up-regulated the mRNA expression levels of the *occludin* gene at 9 dpi, and down-regulated at 1 dpi, 6 dpi, 8 dpi, 10 dpi, 29 dpi, 30 dpi, 35 dpi and 36 dpi. Inhibition of C3aR led to significant up-regulation of the *occludin* gene at 1 dpi, 35 dpi and 36 dpi, but down-regulation from 4 dpi, 6 dpi, 8 dpi, 9 dpi, 10 dpi and 29 dpi (Figure 4C).

### 3.5. Transcriptional Expression Level Analysis of the lgr5 and ki67 in the Ileum Tissues of Mice during C. parvum Infection

**Figure 5:**
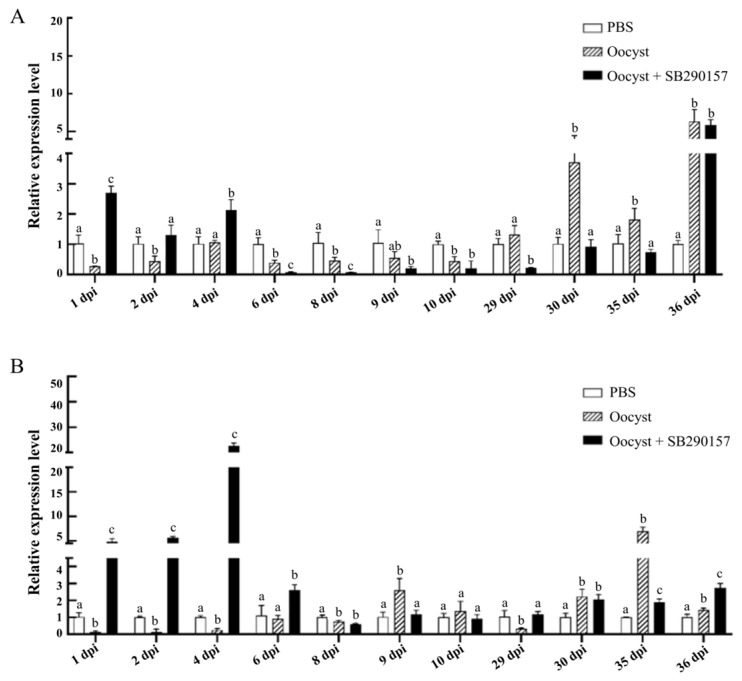
Effect of C3aR inhibition on the relative transcriptional expression levels of the *lgr5* gene (**A**) and *ki67* gene (**B**) in the ileum tissues of mice infected with *C. parvum*. Different lowercase letters above the bars indicated significant differences (*p* < 0.05).

Significant differences were found between the relative transcriptional expression levels of the *lgr5* gene among three groups (PSB, Oocyst, and Oocyst + SB290157) at 1 dpi (F = 115.620, *p* < 0.001), 2 dpi (F = 8.921, *p* = 0.016), 4 dpi (F = 20.221, *p* = 0.002), 6 dpi (F = 41.482, *p* < 0.001), 8 dpi (F = 15.293, *p* = 0.004), 9 dpi (F = 6.992, *p* = 0.027), 10 dpi (F = 16.161, *p* = 0.004), 29 dpi (F = 23.714, *p* = 0.001), 30 dpi (F = 35.947, *p* < 0.001), 35 dpi (F = 11.568, *p* = 0.009) and 36 dpi (F = 25.923, *p* = 0.001). Compared with the control group, the mRNA expression levels of the *lgr5* gene of mouse ileum tissues in the *C. parvum*-infected group were significantly down-regulated at 1 dpi, 2 dpi, 6 dpi, 8 dpi and 10 dpi, and up-regulated from 30 dpi, 35 dpi and 36 dpi. Inhibition of C3aR led to significant up-regulation of the *lgr5* gene at 1 dpi, 2 dpi and 4 dpi, but down-regulation at 6 dpi, 8 dpi, 29 dpi, 30 dpi and 35 dpi (Figure 5A).

Significant differences were found between the relative transcriptional expression levels of the *ki67* gene among three groups (PSB, Oocyst, and Oocyst + SB290157) at 1 dpi (F = 136.699, *p* < 0.001), 2 dpi (F = 621.666, *p* < 0.001), 4 dpi (F = 1282.733, *p* < 0.001), 6 dpi (F = 15.791, *p* = 0.004), 8 dpi (F = 19.258, *p* = 0.002), 9 dpi (F = 10.979, *p* = 0.010), 29 dpi (F = 12.055, *p* = 0.008), 30 dpi (F = 12.545, *p* = 0.007), 35 dpi (F = 119.732, *p* < 0.001) and 36 dpi (F = 69.487, *p* < 0.001), while the difference at 10 dpi (F = 1.225, *p* = 0.358) was not significant. *Cryptosporidium parvum* infection significantly up-regulated the mRNA expression levels of the *ki67* gene at 9 dpi, 30 dpi, 35 dpi and 36 dpi, but down-regulated at 1 dpi, 2 dpi, 4 dpi, 8 dpi and 29 dpi. Inhibition of C3aR led to the significant up-regulation of the *ki67* at 1 dpi, 2 dpi, 4 dpi, 6 dpi, 29 dpi and 36 dpi, but down-regulation at 9 dpi and 35 dpi (Figure 5B).

### 3.6. Transcriptional Expression Level Analysis of CD4^+^ T cell-Related Cytokines in the Ileum Tissues of Mice during C. parvum Infection

**Figure 6:**
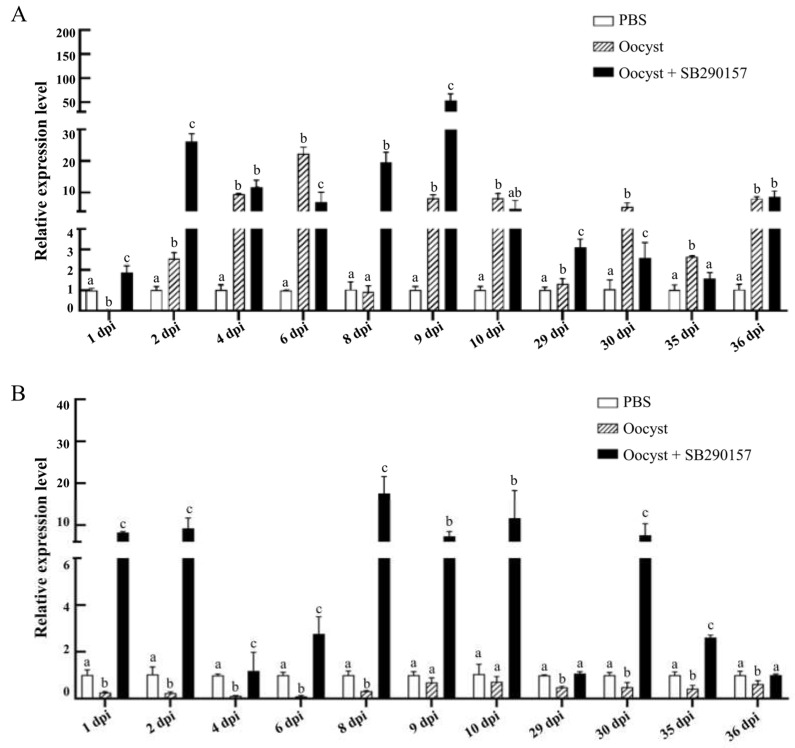
Effect of C3aR inhibition on the relative transcriptional expression of the CD4^+^ T cell-related cytokines in the ilea of mice infected with *C. parvum*. Analysis of the relative expression levels of the *ifn-γ* (**A**) and *tgf-β* (**B**) genes in the ileum tissues of mice. Different lowercase letters above the bars indicated significant differences (*p* < 0.05).

Significant differences were found between the relative transcriptional expression levels of the *ifn-γ* gene among the three groups (PSB, Oocyst, and Oocyst + SB290157) at 1 dpi (F = 69.841, *p* < 0.001), 2 dpi (F = 300.925, *p* < 0.001), 4 dpi (F = 58.746, *p* < 0.001), 6 dpi (F = 72.761, *p* < 0.001), 8 dpi (F = 97.252, *p* < 0.001), 9 dpi (F = 38.113, *p* < 0.001), 10 dpi (F = 12.446, *p* = 0.007), 29 dpi (F = 48.334, *p* < 0.001), 30 dpi (F = 19.592, *p* = 0.002), 35 dpi (F = 41.574, *p* < 0.001) and 36 dpi (F = 45.236, *p* < 0.001). Compared with the control group, the mRNA expression levels of the *ifn-γ* gene of mouse ileum tissues in *C. parvum*-infected group were significantly down-regulated at 1 dpi, but up-regulated at most time points. Inhibition of C3aR led to the significant up-regulation of *ifn-γ* at 1 dpi, 2 dpi, 8 dpi, 9 dpi and 29 dpi, but down-regulation at 6 dpi, 30 dpi and 35 dpi (Figure 6A).

Significant differences were found between the relative transcriptional expression levels of the *tgf-β* gene among three groups (PSB, Oocyst, and Oocyst + SB290157) at 1 dpi (F = 1721.105, *p* < 0.001), 2 dpi (F = 35.969, *p* < 0.001), 6 dpi (F = 30.388, *p* = 0.001), 8 dpi (F = 51.836, *p* < 0.001), 9 dpi (F = 94.612, *p* < 0.001), 10 dpi (F = 8.058, *p* = 0.020), 29 dpi (F = 87.382, *p* < 0.001), 30 dpi (F = 19.085, *p* = 0.003), 35 dpi (F = 205.922, *p* < 0.001) and 36 dpi (F = 8.847, *p* = 0.016), while the difference at 4 dpi (F = 4.687, *p* = 0.059) was not significant. *Cryptosporidium parvum* infection significantly down-regulated the mRNA expression level of the *tgf-β* at most time points. Inhibition of C3aR led to the significant up-regulation of *tgf-β* at all time points post infection (Figure 6B).

## 4. Discussion

Cryptosporidiosis is an important zoonotic disease caused by *Cryptosporidium* spp., which greatly threatens the health of humans and animals, especially young and immunocompromised hosts [1,2]. However, there are still no effective drugs and vaccines for the prevention and control of cryptosporidiosis. One of the main reasons for this is a lack of knowledge on the interaction between *Cryptosporidium* and hosts. Previous studies indicated C3a/C3aR signaling played some important roles in the recovery of intestinal injury [22]. Our previous study showed that *C. parvum* infection could lead to the up-regulation of C3a and C3aR in mouse ileum tissues [24], but the role of C3a/C3aR signaling during *C. parvum* infection is still unknown. The present study applied an optimized mouse model with *C. parvum* infection to explore the function of C3a/C3aR signaling during *C. parvum* infection, enriching our knowledge on the interaction between *Cryptosporidium* and hosts.

The present study optimized an established suckling mouse model infected with *C. parvum* in our group by increasing the concentration of sodium taurocholate and the excystation time, and the results indicated that the optimized model significantly increased the excystation of *C. parvum*. Similar to the previous model, the optimized model also found pathological damage and an inflammatory reaction in the intestines in mice during *C. parvum* infection [31]. Compared with other infection models, the optimized mouse model in the present study simulated the natural infection process of animals and provided a candidate model for studies on the interaction between *Cryptosporidium* and hosts without the use of dexamethasone.

In the present study, the optimized mouse model found that the up-regulation of C3aR in the ileum of mice during *C. parvum* infection and inhibition of C3aR significantly aggravated intestinal damage caused by *C. parvum* infection. Although opposite results were found for the C3aR mRNA expression at 1 dpi and 2 dpi between the present study and our previous work, the mRNA levels of this gene were increased at later stages of infection for both studies [24]. The differences at the early stage of infection may be due to the distinct immunity of animal models. The present study used suckling mice for parasite infection, while older mice aged 3 weeks were used in the previous study.

Tight junctions are an important structural basis of the intestinal mucosal mechanical barrier, which plays an important role in maintaining the integrity of the intestinal morphology and resisting the invasion of intestinal pathogens [33]. To explore the mechanisms of C3a/C3aR signaling, the mRNA expression levels of three tight junction proteins, namely *zo-1*, *claudin 3* and *occludin*, were detected by using qPCR. The results found that *C. parvum* infection down-regulated the mRNA expression levels of the *zo-1*, *claudin 3* and *occludin* genes at most time points, indicating that *C. parvum* likely destroyed the integrity of intestinal barrier by down-regulating tight junctions, which was similar to the C57BL/6 mouse model infected with *C. parvum* [34]. Meanwhile, *Cryptosporidium andersoni* infection could also destroy the expression of the ZO-1 in the epithelial cells of humans and cattle [35]. Interestingly, inhibition of C3aR significantly down-regulated the mRNA expression of the *occludin* gene at half of the time points during *C. parvum* infection, suggesting that C3a/C3aR signaling likely maintained the integrity of the intestinal barrier by upregulating the expression of the tight junction-related gene *occludin*, thus reducing the infection and propagation of *C. parvum* in mouse ilea.

Meanwhile, the proliferation and differentiation of intestinal stem cells and the normal renewal of intestinal epithelial cells also play some important roles in maintaining the structure and function of intestinal mucosa [22,36]. Furthermore, the mRNA expression levels of intestinal stem cell marker LGR5 and cell proliferation marker KI67 of ileum tissues during *C. parvum* infection were detected by qPCR. The results indicated that *C. parvum* infection down-regulated the mRNA expression levels of the *lgr5* and *ki67* genes in mouse ileum tissues at half of the time points, reflecting that *C. parvum* infection possibly retarded the proliferation and renewal of intestinal epithelial cells to prolong the survival time of the parasites in intestinal cells, and then aggravated *Cryptosporidium* infection in intestine, in accordance with previous reports [37,38]. Inhibition of C3aR led to the down-regulation of the *lgr5* gene at most time points, while leading to up-regulation of *ki67* at most time points, suggesting that C3a/C3aR signaling likely promoted the regeneration of intestinal epithelial cells and inhibited the proliferation of intestinal epithelial cells to inhibit the propagation of *C. parvum* in the ilea of mice.

Early moderate IFN-γ can not only initiate intestinal immunity, but can also promote intestinal cell proliferation and mucosal damage repair. However, abnormal over-activation of the immune system will release a large amount of IFN-γ, and excessive IFN-γ can not only kill pathogenic microorganisms, but also activate the JAK/STAT1 pathway of intestinal epithelial cells, leading to programmed necrosis of intestinal epithelial cells and impairment of intestinal barrier function [36]. Unlike IFN-γ, TGF-β showed a protective function on the intestinal barrier [39]. The aforementioned functions of IFN-γ and TGF-β have also been confirmed in intestinal epithelial cells infected with *Cryptosporidium* [40,41,42]. Meanwhile, C3a/C3aR signaling can function in resisting pathogen invasion by promoting/inhibiting the expression of IFN-γ and TGF-β [43,44]. To explore the link between C3a/C3aR signaling and the expression of IFN-γ and TGF-β, we used qPCR to detect the mRNA expression levels of the *ifn-γ* and *tgf-β* genes in mouse ilea during *Cryptosporidium* infection. The results revealed that *C. parvum* infection significantly up-regulated and down-regulated the mRNA levels of the *ifn-γ* and *tgf-β* genes, respectively, indicating that *C. parvum* infection may induce a Th1-type immune response, and inhibit the differentiation and function of Treg cells. Therefore, mice infected with *C. parvum* were likely in a state of continuous inflammation. However, inhibition of C3aR led to the significant up-regulation of mRNA levels of both *ifn-γ* and *tgf-β* in the ileum tissues of infected mice, which was possibly related to the dual anti-inflammatory and pro-inflammatory actions of C3a/C3aR signaling [45], suggesting that C3a/C3aR signaling likely participated in the anti-*Cryptosporidium* effect by down-regulating the expression of Th1 and Treg cells’ main effect factors; however, these mechanisms need to be explored in future studies.

## 5. Conclusions

The present study firstly explored the preliminary function of host C3a/C3aR signaling during *C. parvum* infection, and revealed C3a/C3aR signaling likely inhibited the propagation of *C. parvum* in the ilea of mice by regulating the gut barrier, cell proliferation, and CD4^+^ T cell main effectors of hosts, which could contribute to our knowledge on the interaction between *Cryptosporidium* and hosts.

## Figures and Tables

**Table 1:** Primers for real-time PCR amplification in the present study.

Target Gene	Sequence (5′-3′)	Annealing Temperature (°C)
*18S rRNA*	F: CTCCACCAACTAAGAACGGCC R: TAGAGATTGGAGGTTGTTCCT	55
*gapdh*	F: GGTGAAGGTCGGTGTGAACG	55
	R: CTCGCTCCTGGAAGATGGTG	
*C3aR*	F: CTATTGGGACTGCTAGGCAA R: TGTCCTTGGAGAATCAGGTG	54
*zo-1*	F: GCCGCTAAGAGCACAGCAA R: GCCCTCCTTTTAACACATCAGA	54
*claudin 3*	F: ACCAACTGCGTACAAGACGAG R: CGGGCACCAACGGGTTATAG	55
*occludin*	F: TGAAAGTCCACCTCCTTACAGA R: CCGGATAAAAAGAGTACGCTGG	54
*lgr5*	F: GGCAGCACTTTTCAGCA R: GGACGACAGGAGATTGGA	53
*ki67*	F: TCTGTGCTGACCCTGATG R: CCCTGATGAGTCTTGGCTA	51
*ifn-γ*	F: ATGAACGCTACACACTGCATC R: CCATCCTTTTGCCAGTTCCTC	55
*tgf-β*	F: CCGCAACAACGCCATCTAT R: CCAAGGTAACGCCAGGAATT	55

## Data Availability

Data are contained within the article.

## Supplementary Materials

The following supporting information can be downloaded at: https://www.mdpi.com/article/10.3390/ani13050837/s1, Figure S1: Original Western blot picture for the protein expression level of C3aR in the mouse ileum tissues of PBS and Oocyst groups at 2 dpi; Figure S2: Original Western blot picture for the protein expression level of C3aR in the mouse ileum tissues of PBS and Oocyst groups at 6 dpi; Figure S3: Original Western blot picture for the protein expression level of C3aR in the mouse ileum tissues of PBS and SB290157 groups at three hours after intraperitoneal injection with SB290157.
