# Supplementary material for: C3a/C3aR Affects the Propagation of Cryptosporidium parvum in the Ileum Tissues of Mice by Regulating the Gut Barrier, Cell Proliferation, and CD4+ T Cell Main Effectors
Source: Animals (Basel). 2023 Feb 24;13(5):837. doi: 10.3390/ani13050837 (PMC10000055; doi:10.3390/ani13050837)
Supplement: Supplementary file 1 [file animals-13-00837-s001.zip › animals-2141793-supplementary.pdf]

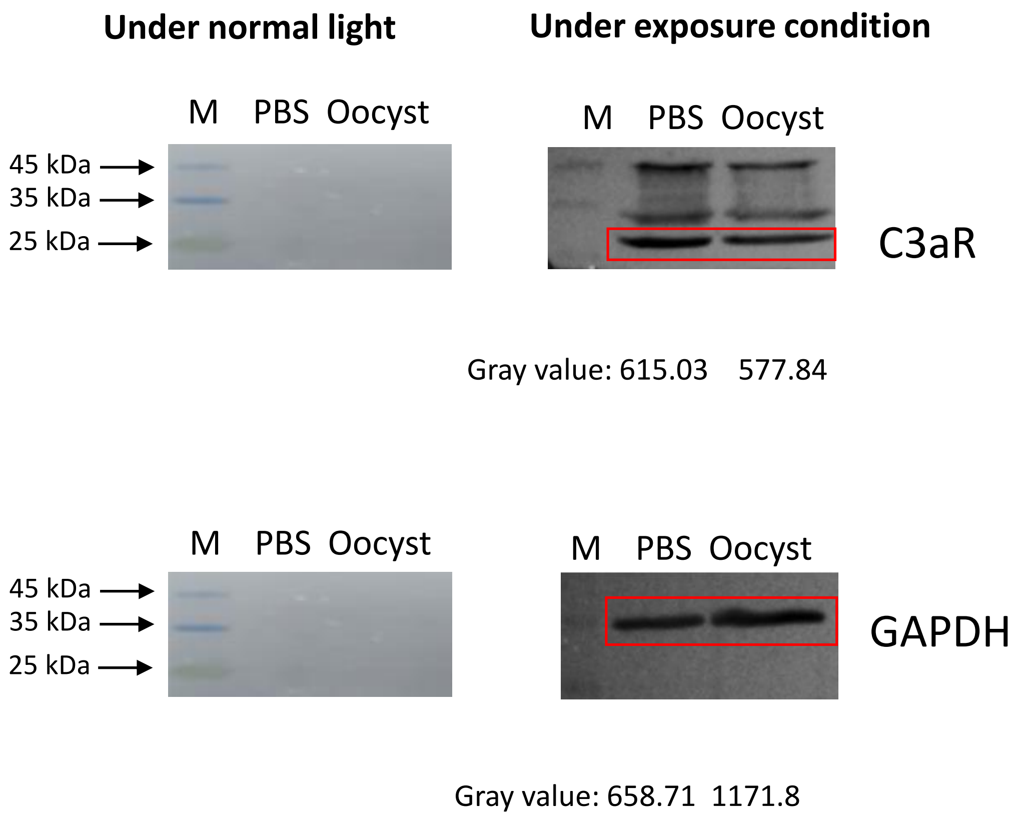

Figure S1. Original Western blot picture for the protein expression level of C3aR in the mouse ileum tissues of PBS and Oocyst groups at 2 dpi.

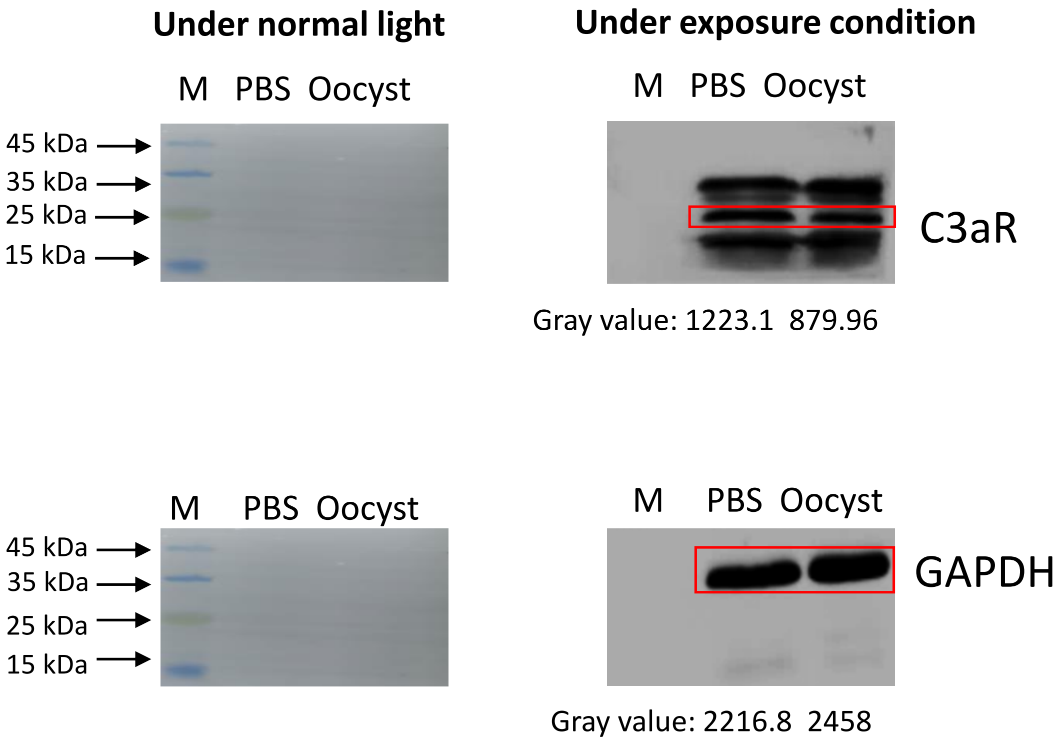

Figure S2. Original Western blot picture for the protein expression level of C3aR in the mouse ileum tissues of PBS and Oocyst groups at 6 dpi.

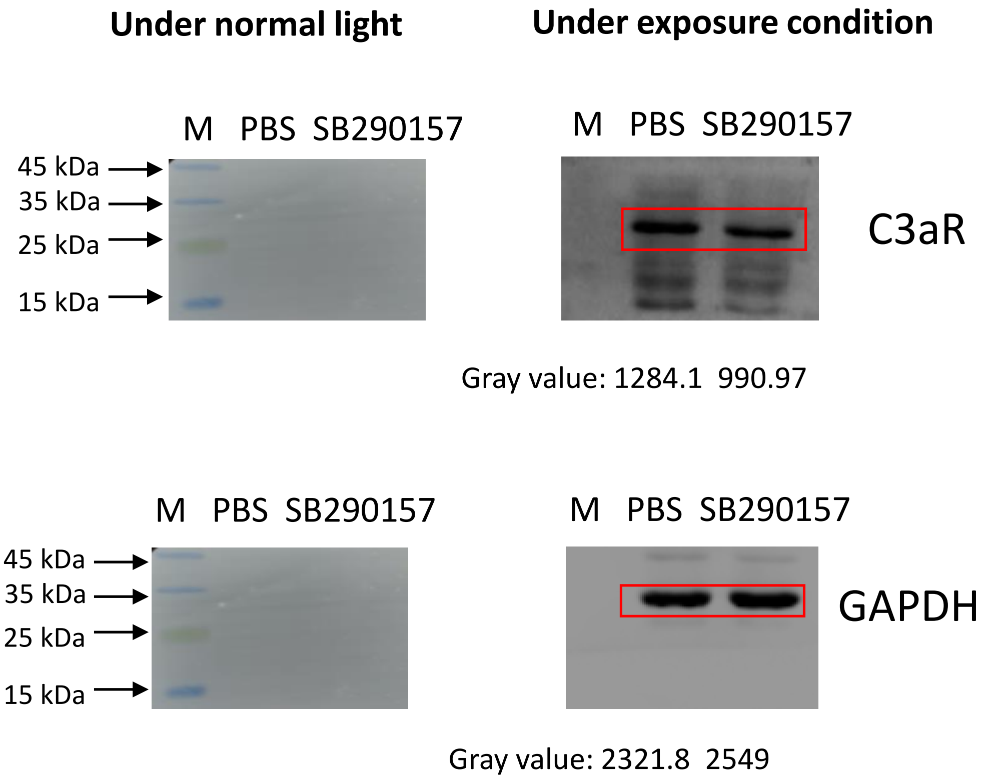

Figure S3. Original Western blot picture for the protein expression level of C3aR in the mouse ileum tissues of PBS and SB290157 groups at three hours after intraperitoneal injection with SB290157.
